# Delivery Mode and Perinatal Antibiotics Influence the Infant Gut Bacteriome and Mycobiome: A Network Analysis

**DOI:** 10.3390/jof9070718

**Published:** 2023-06-30

**Authors:** Mysore V. Tejesvi, Jenni Turunen, Sonja Salmi, Justus Reunanen, Niko Paalanne, Terhi Tapiainen

**Affiliations:** 1 Research Unit of Clinical Medicine, University of Oulu, 90014 Oulu, Finland; 2 Ecology and Genetics, Faculty of Science, University of Oulu, 90014 Oulu, Finland; 3 Biocenter Oulu, University of Oulu, 90014 Oulu, Finlandjustus.reunanen@oulu.fi (J.R.); 4 Research Unit of Translational Medicine, University of Oulu, 90014 Oulu, Finland; 5 Disease Networks Research Unit, University of Oulu, 90014 Oulu, Finland; 6 Department of Pediatrics and Adolescent Medicine, Oulu University Hospital, 90014 Oulu, Finland

**Keywords:** mycobiome, bacteriome, 16S rRNA, delivery mode, intrapartum antibiotics, network analysis, internal transcribed spacer region

## Abstract

Both exposure to antibiotics at birth and delivery via Caesarean section influence the gut bacteriome’s development in infants. Using 16S rRNA and internal transcribed spacer sequencing on the Ion Torrent platform, we employed network analysis to investigate the bacterial and fungal interkingdom relationships in the gut microbiome from birth to age 18 months in a prospective cohort study of 140 infants. The gut microbiome at ages six and 18 months revealed distinctive microbial interactions, including both positive and negative associations between bacterial and fungal genera in the gut ecosystem. Perinatal factors, delivery mode and intrapartum antibiotic exposure affected the associations between bacterial and fungal species. In infants exposed and unexposed to perinatal antibiotics, the gut microbiome formed distinct networks for the bacteriome and mycobiome. The fungi *Saccharomyces*, *Trichosporon*, *Pezoloma*, *Cystofilobasidium*, *Rigidoporus* and *Fomitopsis* were strongly associated with exposure to antibiotics at birth. *Hyaloscypha*, *Trichosporon*, *Fomitopsis* and *Vishniacozyma* were strongly associated with the control group that was not exposed to antibiotics. Five distinct networks were formed according to delivery mode. The present study confirms that bacteria and fungi clearly interact in the infant gut ecosystem. Furthermore, perinatal factors appear to influence the relationships between bacteria and fungi in the developing gut microbiome.

## 1. Introduction

The human gastrointestinal tract is home to a diverse population of microorganisms that includes not only bacteria but also communities of fungi, archaea and viruses [1,2,3]. Fungi constitute approximately 0.1% of the human microbiome [4,5,6]. Mycobiomes are currently being studied for their potential impact on human health and disease, although the precise role played by fungi is yet unknown [4,5,6]. In infancy, the gut microbial populations evolve concurrently, including the bacteriome and mycobiome [7].

The estimation of microbe-microbe association networks [8,9,10] is an exploratory research method for microbiome data [11,12] that provides high-level insights into the overall organisation of microbial communities. A single microbial association network may provide an understanding of the basic organisational structure of a microbial community. For instance, statistical network analysis tools that ignore the compositional structure of data may inspire false conclusions, often referred to as compositional effects [13]. To avoid such errors, researchers may use correlation estimators, such as SparCC [14] and partial correlation estimators [15,16], as well as tools that measure and estimate microbial relationships while taking compositional considerations into account [17]. Previously, we have shown how antibiotic exposure at birth influences the development of bacterial communities in the gut microbiome [18,19,20] and have characterised the development of the gut mycobiome in a prospective cohort study of healthy infants [21]. Limited data exist, however, on the interplay of the bacteriome and mycobiome in gut colonisation in children.

The analysis of the association between fungi and bacteria in the infant gut microbiome was conducted due to the limited understanding of their interactions, and early life microbial colonization plays a crucial role in immune system development, metabolism and overall health. The present study employed network analysis to examine the interkingdom interactions between various genera of the bacterial and fungal kingdoms during the maturation of the gut microbiome from birth to infancy in a prospective child cohort study.

## 2. Materials and Methods

### 2.1. Study Design and Oversight

This prospective cohort study of 152 newborn infants examined the impact of microbial networks during the maturation of the gut bacteriome and mycobiome between birth and 18 months of age. A portion of the meconium samples used in previous studies was not included in this analysis; thus, they were unavailable for the network analysis conducted in this study. The protocol was evaluated and approved by the ethical committee of the Northern Ostrobothnia Hospital District at Oulu University Hospital, Finland (Decision 3/2016). The families provided written informed consent in advance, and the study was conducted in accordance with applicable regulations and standards. In all, 429 samples were collected, all of which were used for mycobiome and 415 for bacteriome analysis, with the sample donors ranging in age from birth to 18 months (Table 1). We used 50 negative control samples (HyClone HyPure, Thermo Fisher Scientific, Waltham, MA, USA) alongside the faecal samples to control for environmental contamination in the analyses.

### 2.2. Sample Collection

Samples of the first-pass meconium were collected from the diaper after birth by a midwife in the delivery room or a nurse on the ward. The stool samples from young children were obtained at home by the families at six and 18 months. All the samples were delivered to the hospital laboratory and stored at −80 °C until further processing.

### 2.3. DNA Extraction

A DNeasy PowerSoil Pro Kit (Qiagen, Hilden, Germany) was used to extract DNA from the meconium and stool samples, following the manufacturer’s instructions. Each 200 mg sample was weighed out and mixed with 1 mL of phosphate buffer saline (PBS). The samples were homogenised by bead beating with a TissueLyser (Qiagen) at a rate of 25 Hz for two minutes, followed by a one-minute incubation period on ice. The homogenisation was repeated one to three times. Instead of using the TissueLyser, we processed the negative control samples and minimal faecal material samples according to the vortex adapter homogenisation methodology. After homogenisation, the final elution was adjusted to 100 mL, and extraction was conducted using a QIAcube Connect extraction device (Qiagen). A NanoDrop 1000 spectrophotometer was used to measure DNA concentration and quality (Thermo Fisher Scientific).

### 2.4. PCR, Sequencing and Analysis

#### 2.4.1. Bacteriome

The polymerase chain reaction (PCR) and the sequencing of the bacterial 16S gene of the meconium samples were performed at the DNA Sequencing and Genomics Laboratory, Institute of Biotechnology, University of Helsinki. A Phusion HotStart enzyme was used in the PCR reaction. Beginning with the first PCR, a two-step process was followed using a mixture of the primers 341F (5′ CCTACGGGNGGCWGCAG 3′) and 785R (5′ GACTACHVGGGTATCTAATCC 3′). The overhangs in the first PCR were targeted in the second PCR using dual-index primers that were chosen using BARCOSEL [22]. The ZymoBIOMICS Microbial Community DNA Standard was used as a positive control for the sequencing study. Using a 600-cycle v3 sequencing kit on MiSeq (Illumina, San Diego, CA, USA), the PCR products were pooled, purified and paired-end sequenced. The children’s six- and 18-month stool samples were sequenced for the full-length 16S rRNA gene using PacBio sequencing technology at the DNA Sequencing and Genomics Laboratory, Helsinki. The full-length 16S rRNA gene was amplified using primers 27F (5-AGAGTTTGATCMTGGCTCAG-3) and 1492R (5-GGTTACCTTGTTACGACTT-3). The PacBio RS II sequencing machine (Pacific Biosciences, Menlo Park, CA, USA) was used to sequence approximately 1465 bp. The 16S rRNA gene sequences were processed in the exact manner of shorter MiSeq reads with the maximum length set to 1465 bp.

#### 2.4.2. Mycobiome

The primers fITS7b (5′-G TGARTCATCGAATCTTTG-3′) and ITS4 (5′-TCCTCCGCTTATTGATATGC-3′) with distinctive barcodes were used to sequence the internal transcribed spacer 2 (ITS2) region at the University of Oulu. Phusion Flash High-Fidelity PCR master mix (Thermo Fisher Scientific) was used to perform PCR, following the manufacturer’s instructions. The PCR and sequencing methods are described in detail elsewhere [21].

### 2.5. Bioinformatics Analysis

For first-pass meconium samples, QIIME2 (versions 2021.2 and 2022.8) was used to conduct the downstream analysis [23]. The reads were demultiplexed after being imported into the paired-end Phred+33 format. DADA2 [24] was used to denoise and filter chimeras. For forward reads, the reads were clipped at base 15 and truncated at base 280; reverse reads were truncated at base 220. The results of sequence-based microbiome analyses are sensitive to contamination [25], so any reagent and sample contaminations were eliminated through the use of negative controls and the R program’s decontam package (version 1.16.0), which uses a prevalence-based approach and a threshold of 0.5 [26]. In the QIIME2 analysis, taxa categorised as Mitochondria, Eukaryota, Cyanobacteria and Archaea were not included.

For the mycobiome analysis, sequences that were less than 200 bp were discarded. Using DADA2, the sequence data were demultiplexed and denoised [24]. Chimeric reads were identified and removed, and reads were cut at bases 15 and 160. The decontam package was used similarly for the mycobiome data, as described above. The gut bacteriome and mycobiome data were rarefied to a depth of 2006 prior to merging. For alpha and beta diversity, the reads were based on the lowest read count of the sample.

### 2.6. Network Analysis

The microbial network was studied using Pearson correlation networks and the Netcomi package in R (version 4.1.0). Using Compare () within the Netcomi package, the delivery mode and antibiotic usage were determined between the groups. Features present in less than 5% of the samples were removed before analysis. The combined count matrix was provided to netConstruct () for network comparison, and a distinct binary vector assigning samples to the antibiotic and control groups was included. The association between antibiotics and delivery mode groups was measured using the SPRING method. The predicted partial correlations were transformed into dissimilarities via the ‘signed’ distance metric, and the corresponding similarities were then used as edge weights [17].

### 2.7. Data Availability

The raw sequences with the BioProject identifiers PRJNA905086, PRJNA935710 and PRJNA831656 have been submitted to GenBank.

## 3. Results

### 3.1. Microbial Network Analysis

We used Pearson association correlations to evaluate the sparse genus-genus association networks for meconium, six-month stool, 18-month stool and the entire population to assess the possible changes in the organisational structure during the maturation of the infant gut bacteriome and mycobiome.

**Figure 1:**
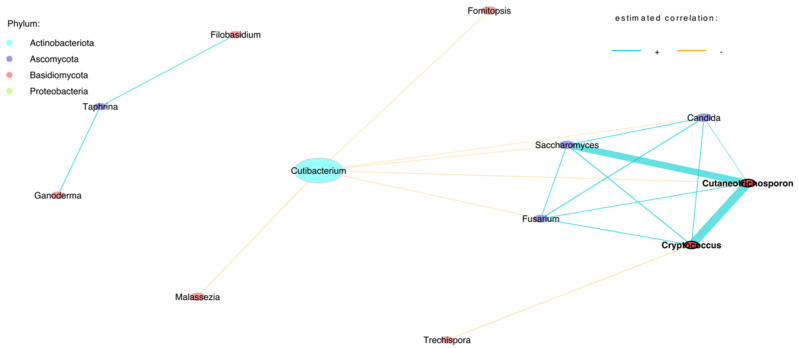
Genera-level network of merged bacteriome and mycobiome data of meconium samples.

Firstly, we used all samples obtained at birth to characterise the overall microbial structure using Pearson association networks (Figure 1). Each network had two sparsely linked components comprising both mycobiome and bacteriome genera. These networks included the majority of previously identified potentially abundant taxa, such as the bacterium *Cutibacterium* and the fungi *Saccharomyces*, *Candida*, *Fusarium*, *Cutaneotrichosporon*, *Cryptococcus*, *Malassezia* and *Trechispora. Cutibacterium* was negatively associated with the fungi *Saccharomyces*, *Candida* and *Fusarium*, whereas *Saccharomyces* was positively associated with *Cutaneotrichosporon* and *Cryptococcus.* Furthermore, a separate network was formed by *Filobasidium*, *Taphrina* and *Ganoderma* (Figure 1).

**Figure 2:**
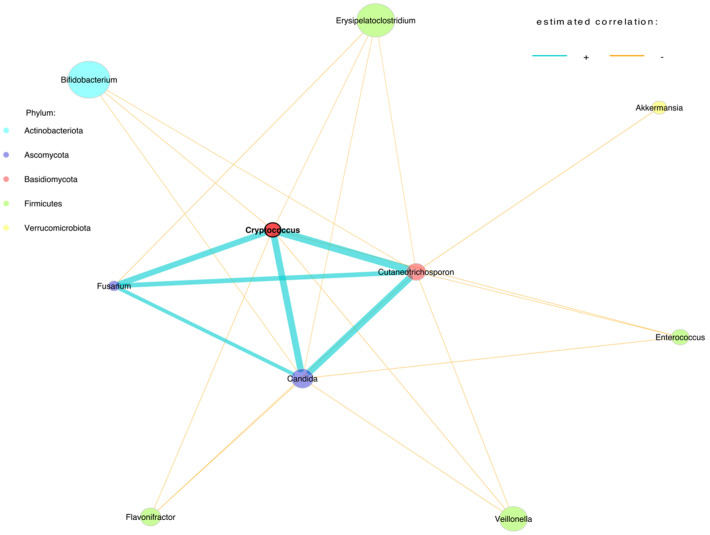
Genera-level network of merged bacteriome and mycobiome data of stool samples at six months.

In the six-month faecal samples, we observed positive associations between *Candida*, *Fusarium*, *Cryptococcus* and *Cutaneotrichosporon* and negative associations between *Bifidobacterium*, *Erysipelatoclostridium*, *Akkermansia*, *Enterococcus*, *Flavonifractor* and *Veillonella* (Figure 2).

**Figure 3:**
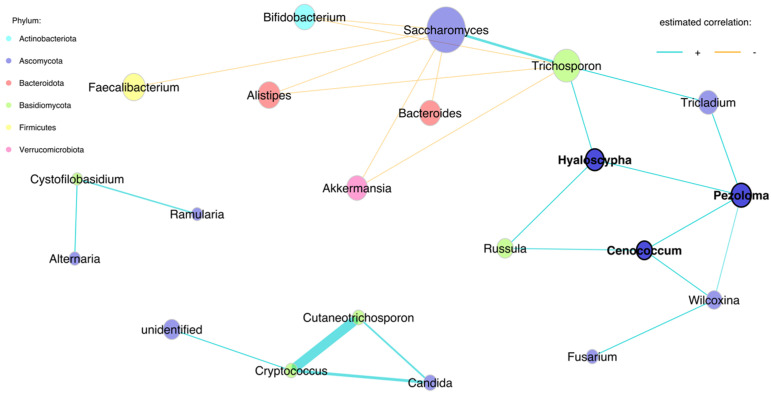
Genera-level network of merged bacteriome and mycobiome data of stool samples at 18 months.

**Figure 4:**
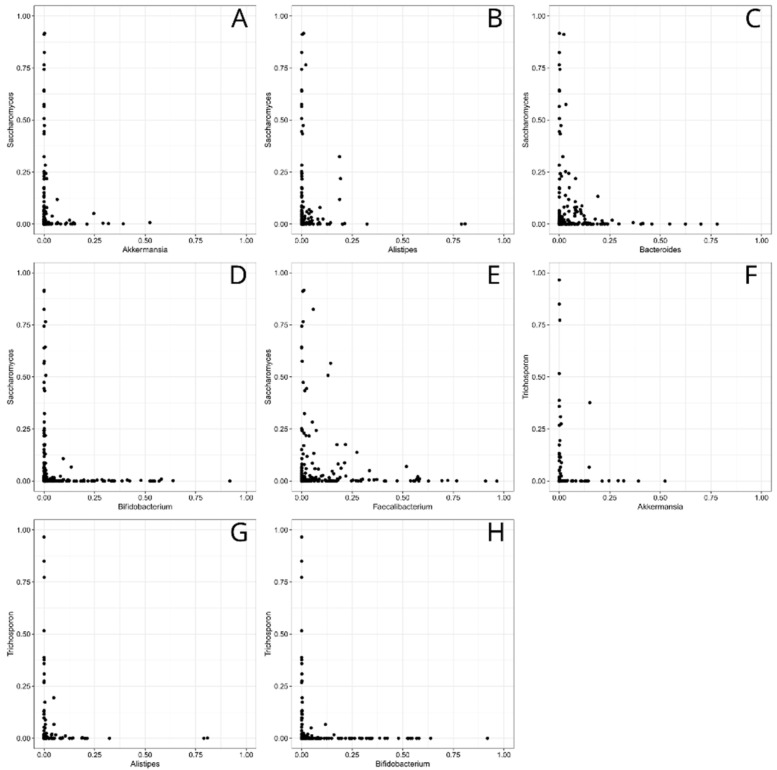
The negative associations between the abundances of (**A**) *Saccharomyces* and *Akkermansia*; (**B**) *Saccharomyces* and *Alistipes*; (**C**) *Saccharomyces* and *Bacteroides*; (**D**) *Saccharomyces* and *Bifidobacterium*; (**E**) *Saccharomyces* and *Faecalibacterium*; (**F**) *Trichosporon* and *Akkermansia*; (**G**) *Trichosporon* and *Alistipes*; and (**H**) *Trichosporon* and *Bifidobacterium*. The horizontal axis represents the relative abundances of bacteria, while the vertical axis represents the relative abundances of fungi.

We found one large and two small networks in the 18-month stool samples (Figure 3). In the large network, *Trichosporan*, *Tricladium*, *Hyaloscypha*, *Pezoloma*, *Cenococcum*, *Russula*, *Wilcoxina* and *Fusarium* were positively associated with *Saccharomyces.* A positive association was found between the genera *Cryptococcus*, *Candida*, *Cutaneotrichosporon* and unidentified fungi. *Faecalibacterium*, *Bifidobacterium*, *Alistipes and Akkermansia* were negatively associated with *Saccharomyces* and *Bacteroides. Bifidobacterium*, *Alistipes* and *Akkermansia* had a negative association with *Trichosporon* (Figure 3). The negative associations between the abundances of *Saccharomyces* with *Akkermansia*, *Alistipes*, *Bacteroides*, *Bifidobacterium* and *Faecalibacterium*, including *Trichosporon* with *Akkermansia*, *Alistipes* and *Bifidobacterium*, are shown in Figure 4.

### 3.2. Network Comparative Analysis according to Perinatal Factors in All Age Groups

**Figure 5:**
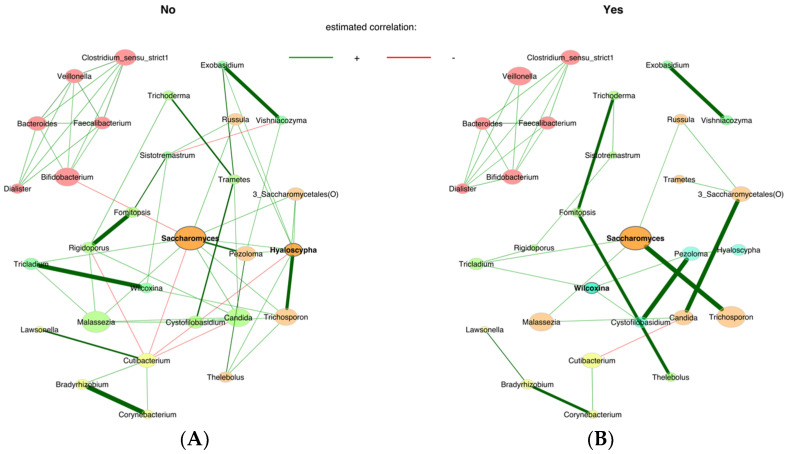
Associations between the bacteriome and mycobiome according to perinatal factors. The association measure employed was the SPRING method. Red edges denote negative estimated associations, whereas green edges represent positive ones. (**A**): Unexposed to antibiotics (**B**): Exposed to intrapartum antibiotics.

We used network analysis in the infant groups according to perinatal factors from birth to 18 months only in vaginally delivered samples. In the intrapartum antibiotic group (Figure 5A), strong positive associations were seen between *Saccharomyces* and *Trichosporon*, *Pezoloma* and *Cystofilobasidium* and *Candida* and *Saccharomycetales.* A positive association was seen of *Rigidoporus* with *Tricladium* and *Sistotremastrum* and of *Fomitopsis* with *Trichoderma*, *Thelebolus* and *Cystofilobasidium.* Interestingly, *Exobasidium* and *Vishniacozyma* formed a distinct network. In the group not exposed to antibiotics, *Rigidoporus*, *Exobasidium*, *Saccharomyces* and *Hyaloscypha* had strong positive associations with *Fomitopsis*, *Vishniacozyma*, *Pezoloma* and *Trichosporon*, respectively (Figure 5B). Moreover, *Tricladium* with *Wilcoxina* and *Bradyrhizobium* with *Corynebacterium* showed positive associations.

**Figure 6:**
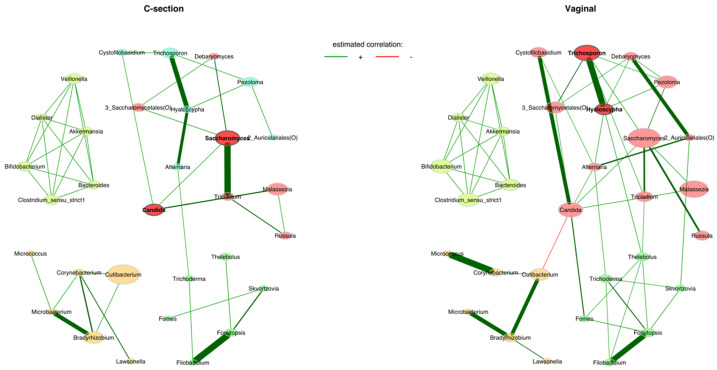
Associations between the bacteriome and mycobiome according to the delivery modes Caesarean section and vaginal birth. The association measure employed was the SPRING method. Red edges denote negative estimated associations, whereas green edges represent positive ones.

We found three networks in the Caesarean section group and two networks in the vaginally delivered infants (Figure 6). In infants born via Caesarean section, *Hyaloscypha* was positively associated with *Trichosporon* and *Alternaria*, *Microbacterium* with *Bradyrhizobium*, *Filobasidium* with *Fomitopsis* and *Saccharomyces* with *Tricladium*. In the vaginally born infant group, strong positive associations were seen between *Trichosporon* and *Hyaloscypha*; *Cystofilobasidium* and *Candida*; *Micrococcus* and *Corynebacterium*; *Filobasidium* and *Fomitopsis*; *Debaryomyces* and Auriculariales (O) and *Bradyrhizobium* with *Cutibacterium* and *Microbacterium*. Further, the genus Candida was negatively associated with *Cutibacterium*.

## 4. Discussion

This prospective cohort study identified interkingdom bacterial-fungal associations during the maturation of the gut microbiome from birth until the age of 18 months. The maturity of bacterial-fungal networks increased over time, with a more comprehensive network at 18 months of age. Several positive and negative associations were found in the interkingdom network analysis. Exposure to intrapartum antibiotics and Caesarean section appears to influence the structure of bacterial-fungal associations.

The interactions of the gut bacteriome and mycobiome are important in understanding the complexity of the early development of the infant gut microbiome. There are limited previous data on the interkingdom networks in the gut microbiome. Adults with mental health disorders [27], patients with *Clostridium difficile* infection [28] and immunocompromised patients [29] have been reported to have altered gut bacterial-fungal interkingdom associations. Animal research indicates that early life interkingdom interactions may have a role in long-term health effects [7,30]. Boutin and colleagues found that the mycobiome and bacteriome in the neonatal murine gut interact to affect the severity of allergic airway disease later in life [31].

The association between fungi and bacteria in microbiome studies is a dynamic and evolving field that has gained significant attention in recent years. There is a reciprocal relationship between fungi and bacteria in the gut microbiome; specifically, certain bacterial taxa can influence the composition and function of fungal communities, highlighting the dynamic interplay between bacteriome and mycobiome [32]. Bacterial metabolites were shown to have the ability to modulate fungal growth and virulence, underscoring the significance of considering these interactions for a comprehensive understanding of oral health [33]. The interkingdom crosstalk between fungi and bacteria in the skin microbiome and was found that volatile compounds produced by bacteria can have either inhibitory or promotive effects on fungal growth, indicating a complex relationship between these microorganisms in maintaining skin homeostasis [34]. The relationship between fungi and bacteria in the gut microbiome of infants has not been extensively investigated.

In the current study, we observed that meconium, six- and 18-month samples had different bacterial and fungal community compositions. These findings corroborate the notion of a niche-specific bacteriome and mycobiome formation, which is supported by several human studies, including ones in infants [35,36]. The delivery mode significantly influences the initial gut colonisation in infants and young children. Vaginally delivered infants exhibit microbiota enriched in *Bifidobacteria*, *Escherichia* and *Bacteroides* genera, whereas those born via Caesarean delivery tend to have genera more commonly associated with healthcare environments [31,32,33,34]. Over time, vaginally delivered children have less diversity in their gut microbiomes, whereas infants delivered via Caesarean section display a more varied gut microbiome. In the present study, we extend these findings to the altered networks of bacterial and fungal communities during the maturation of the gut microbiome according to delivery mode and exposure to antibiotics at birth.

The use of anti-fungal and anti-bacterial treatments often induces gut dysbiosis and affects the interaction between the mycobiome and bacteriome. In mouse models, it has been observed that intact microbiota provide resistance against pathogenic fungi colonization compared to animals treated with antibiotics [37,38]. *Enterococcus faecalis* was found to predominate over Lactobacillus in the presence of C. albicans, suggesting a competitive relationship [39,40]. Anti-fungal treatments in mice led to decreased fungal diversity but increased bacterial diversity, aggravating colitis and promoting the growth of pathogenic bacterial microbiota [41]. The intricate interplay between the mycobiome and microbiome, as well as the impact of anti-bacterial and anti-fungal compounds on the gut flora, is emphasized. There have been limited data on the interaction of mycobiome and bacteriome in the developing gut microbiome in infants before the present study.

This study has several strengths. We prospectively used the faecal samples from a large child cohort for bacteriome and mycobiome network analysis to characterise changes during the maturation of the gut microbiome. For samples collected at six and 18 months, we employed full-length 16S gene sequencing, which allowed an accurate taxonomic classification using the SILVA database up to the species level. The data from the bacteriome and mycobiome were merged for network analysis to examine their associations. The study also has limitations. For the meconium and mycobiome samples, we employed partial 16S rRNA and ITS2 spacer sequences, which do not provide species-level taxonomic classification.

## 5. Conclusions

In conclusion, combining bacteriome and mycobiome data helps us better understand their relationship within the gut ecosystem. The network analysis in this prospective study of infants and young children identified positive and negative associations between fungal and bacterial genera in the gut ecosystem. Immediate perinatal factors were shown to alter the associations between bacterial and fungal species, underscoring the potential long-term effects of perinatal events on the developing gut microbiome.

## Figures and Tables

**Table 1:** Number of infants with available faecal samples according to delivery mode and exposure to intrapartum antibiotics during vaginal birth. Samples were obtained at birth (first-pass meconium sample), at six months and at 18 months of age.

**Mycobiome Analyses**			
**Perinatal Factors**	**At Birth**	**Six Months**	**18 months**
Vaginal delivery and no exposure to antibiotics	56	59	59
Vaginal delivery and intrapartum antibiotics	24	33	23
Caesarean delivery	60	60	60
Total	140	152	142
**Bacteriome Analyses**			
**Perinatal Factors**	**At Birth**	**Six Months**	**18 months**
Vaginal delivery and no exposure to antibiotics	46	59	59
Vaginal delivery and intrapartum antibiotics	21	33	23
Caesarean delivery	54	60	60
Total	121	152	142

## Data Availability

The raw Sequences with the Bioproject identifier PRJNA905086, PRJNA935710 and PRJNA831656 were submitted to Genbank.
